# STAT3 drives the malignant progression of low-grade gliomas through modulating the expression of STAT1, FOXO1, and MYC

**DOI:** 10.3389/fmolb.2024.1419072

**Published:** 2024-06-14

**Authors:** Yan Li, Fanjing Jiang, Suhua Zhu, Hongwei Jia, Changwei Li

**Affiliations:** Department of Pharmacy, The Affiliated Xuzhou Municipal Hospital of Xuzhou Medical University, Xuzhou First People’s Hospital, Xuzhou, China

**Keywords:** LGG, STAT3, FoxO1, STAT1, prognosis

## Abstract

Low-grade glioma (LGG) is a prevalent and lethal primary brain malignancy, with most patients succumbing to recurrence and progression. The signal transducer and activator of transcription (STAT) family has long been implicated in tumor initiation and progression. However, a comprehensive evaluation of the expression status and overall function of STAT genes in LGG remains largely unreported. In this study, we investigated the association between the expression of STAT family genes and the progression of LGG. Through a comprehensive analysis that combined bioinformatics screening and validation assays, we determined that STAT1, STAT3, and STAT5A were upregulated and contributed to the malignant progression of LGG. Notably, our findings suggest that STAT3 is a critical prognostic marker that regulates the progression of LGG. STAT3 emerged as the most significant prognostic indicator governing the advancement of LGG. Additionally, our inquiry into the STAT3-binding proteins and differentially expressed-correlated genes (DEGs) revealed that STAT3 played a pivotal role in the progression of LGG by stimulating the expression of STAT1, FOXO1, and MYC. In summary, our recent study conducted a thorough analysis of the STAT family genes and revealed that directing therapeutic interventions towards STAT3 holds potential as a viable strategy for treating patients with LGG.

## Introduction

Gliomas are the most prevalent tumors in the central nervous system, exhibiting high morbidity and mortality rates (Ostrom et al., 2021; Kang et al., 2024). Low-grade glioma (LGG), classified as World Health Organization (WHO) Grade I, II, and III glioma, encompasses astrocytomas, oligodendrogliomas, and oligoastrocytomas (Xu et al., 2019). In general, LGG patients experience a longer median overall survival period compared to those with Glioblastoma (GBM, WHO grade IV) (Xu et al., 2019). Despite advances in treatment with surgery, radiotherapy, and chemotherapy, LGG remains incurable and often progresses to secondary malignant transformation (Bao et al., 2022). Therefore, the diagnosis, monitoring, and treatment of LGG patients pose significant challenges due to the diffuse infiltration of tumor cells and their resistance to radiotherapy and chemotherapy (Hayes et al., 2018).

With the concept of precision medicine, targeted therapy and immune therapy have been proposed and used in the treatment of LGG (Bao et al., 2022; Haddad et al., 2022; Lim, 2022; Lucke-Wold et al., 2024). Drugs targeting cyclin-dependent kinase 9 (CDK9), programmed cell death protein-1 (PD-1), and other proteins have been used in clinical trials to treat glioma (Lucke-Wold et al., 2024). Despite the numerous advancements in targeted therapy and immune therapy for LGG that have been developed in the past decades, the definitive improvement in clinical diagnosis and treatment remains limited and insufficient (Haddad et al., 2022; Lim, 2022; Lucke-Wold et al., 2024). The widely poor prognosis of LGG can be attributed to the great genetic heterogeneity in their clinical behavior (Wang and Mehta, 2019; Ryall et al., 2020). Although various molecular biomarkers, including isocitrate dehydrogenase 1 (IDH1), O-6-methylguanine-DNA methyltransferase (MGMT), ADP ribosylation factor like GTPase 9 (ARL9), and chromosome arms 1p/19q deletion, have been developed, the clinical impact of these biomarkers remains to be fully elucidated (Karpel-Massler et al., 2019; van der Voort et al., 2019; Mathur et al., 2020; Tan et al., 2020). Significant advancements in comprehending the molecular intricacies of LGG have not translated into substantial improvements in the early detection and prognostication of this ailment. Consequently, the development of novel and dependable biomarkers and therapeutic targets for LGG is undeniably imperative and requires immediate attention. The STAT family of proteins, consisting of seven members (STAT1, STAT2, STAT3, STAT4, STAT5a, STAT5b, and STAT6), function as transcription factors that are crucial in various physiological cellular processes, including signal transduction from the cell membrane to the nucleus, regulation of cell proliferation, differentiation, and apoptosis (Turkson and Jove, 2000; Butturini et al., 2020; Verhoeven et al., 2020).

Accumulated research has demonstrated that the abnormal expression of members of the STAT family is associated with cancer cell transformation, metastasis, survival, and resistance to drug treatment (Calò et al., 2003; Loh et al., 2019). Certain STATs, such as STAT3 and STAT5, are considered oncogenes in a variety of tumors, including hematologic and solid tumors (Lassmann et al., 2007; Benekli et al., 2009; Gu et al., 2010). Recent studies have indicated that activated STAT1 functions as a tumor suppressor through the phosphorylation of Tyr701, and the loss of STAT1 activation or expression has been observed in malignant cells (Meissl et al., 2017). Nevertheless, certain investigations have yielded inconsistent findings, indicating that patients with elevated levels of STAT1 expression in cancerous tissues experience inferior clinical outcomes relative to those with lower levels (Khodarev et al., 2010; Magkou et al., 2012; Chou et al., 2022). Khodarev *et al.* regards that activation of STAT1 pathway in breast tumors confers a poor prognosis for patients (Khodarev et al., 2010). Studies have demonstrated that overexpression of phosphorylated STAT1 promotes advanced progression and poorer survival in invasive breast cancer (Magkou et al., 2012). STAT1 has been shown to play an oncogenic role in Colorectal cancer (Chou et al., 2022). This implies that STAT1 may also play a role in tumorigenesis. Conversely, STAT2 is known to exert tumor-suppressive effects through its involvement in anti-apoptotic and anti-proliferative mechanisms in certain tumors (Wang et al., 2003; Du et al., 2009; Yue et al., 2015). Gamero *et al.* have posited that STAT2 may also play a role in the tumorigenesis of colorectal and skin cancers (Gamero et al., 2010). Their hypothesis suggests that STAT2 may activate the oncogenic STAT3 signaling pathway, thereby promoting tumor growth. A body of literature has demonstrated that elevated levels of STAT3 expression are implicated in the development and progression of various cancers, including acute myeloid leukemia, multiple myeloma, and solid tumors affecting the breast, brain, colon, esophagus, head-and-neck, kidney, liver, lung, pancreas, and prostate (Bar-Natan et al., 2012; Lee et al., 2019).

Moreover, STAT3 has been identified as a significant oncogene responsible for the advancement of glioma tumors, and its prevalent expression in high-grade glioma (HGG) is linked to unfavorable clinical outcomes (Doucette et al., 2012). In gastric cancer, the suppression of STAT4 resulted in the inhibition of cell proliferation, migration, and invasion, indicating that STAT4 could be a promising therapeutic target for the treatment of this malignancy (Zhou et al., 2014). Analogously, similar to STAT3, the malfunctioning of inhibitory signaling pathways that regulate STAT5 activation is implicated in the pathogenesis of diverse cancers (Halim et al., 2020). Recent research has revealed a strong correlation between elevated STAT6 expression and unfavorable clinical outcomes in various types of cancer, including breast, gastric, and prostate cancer (Binnemars-Postma et al., 2018; Liu et al., 2019). These findings suggest that STAT genes hold potential as both prognostic indicators and therapeutic targets for cancer treatment. Nevertheless, a comprehensive investigation into the expression patterns and overall functionality of STAT genes in LGG remains largely unexplored.

In this study, we conducted a comprehensive analysis utilizing bioinformatics and validation assays to investigate the impact of STAT family members on the malignant progression of LGG. Our findings indicate that overexpression of STAT1, STAT3, and STAT5A contribute to the progression of LGG. Conversely, STAT4 and STAT6 were found to promote glioma progression by inhibiting the antitumor immune response. However, the effects of STAT2 and STAT5B on the malignant progression of LGG were not found to be statistically significant. Among the members considered, STAT3 emerged as the most significant prognostic signature governing the progression of LGG. Furthermore, our investigation of the STAT3-binding proteins and the differentially expression-correlated genes (DEGs) revealed that STAT3 played a pivotal role in regulating the progression of LGG by upregulating the expression of STAT1, FOXO1, and Myc. In summary, our recent study conducted a comprehensive analysis of the STAT family genes and established that targeting STAT3 represents a promising therapeutic approach for patients with LGG.

## Materials and methods

### Oncomine database analysis of the expression of STAT family in pan-cancer

The Oncomine online cancer gene expression data website (Oncomine Login, www.ocommine.ORG) was utilized to analyze microarray information and a collection of bioinformatic data (Kuang et al., 2022). Specifically, the mRNA expression of STATs in clinical cancer samples was compared with samples in normal sourced from the Oncomine database. Nineteen cancer types and other cancers were used to evaluate and analyze. Amony them, 463 samples were used to analyze the differential expression of STAT1, 426 samples were used to analyze the differential expression of STAT2, 452 samples were used to analyze the differential expression of STAT3, 445 samples were used to analyze the differential expression of STAT4, 415 samples were used to analyze the differential expression of STAT5A, 462 samples were used to analyze the differential expression of STAT5B, and 448 samples were used to analyze the differential expression of STAT6. The resulting *p*-value was determined through the application of one way ANOVA. The statistical significance of the findings was established through the identification of a *p*-value of 0.05 and a folding change of 2.0.

### Analysis of the expression of STAT family in LGG

Firstly, the mRNA expression levels of STAT family genes were assessed in LGG tissues and normal samples through the utilization of the Gene Expression Profiling Interactive Analysis (GEPIA) two online platform (cancer-pku.cn), which incorporated expression data from the TCGA and GTEx databases. Additionally, tumor RNA sequence data was obtained from the genome data sharing (GDC) data portal (https://portal.gdc.cancer.gov/), and the expression of STAT family genes in tumors and normal tissues was subjected to a Wilcox test using R software v4.0.3. Then, the mRNA expression data for 248 grade 1 samples, 261 grade 2 samples, and 2,642 normal samples in LGG were obtained through GDC. Subsequently, Kruskal Wallis one-way ANOVA was conducted on each gene of the STAT family using R software v4.0.3. Statistical significance was determined at *p* < 0.05. The representative protein immunohistochemistry (IHC) staining images for each gene of the STAT family were obtained from LGG and normal tissues in the human protein atlas (HPA) (https://www.proteinatlas.org/).

### Prognosis and diagnostic analysis of STAT family genes in LGG

The present study utilized the Kaplan-Meier Plotter (http://www.kmplot.com/) to assess the impact of STAT mRNA expression on patient survival. The samples were categorized into high and low expression groups based on the median expression of each gene in LGG. The overall survival and disease-free survival were evaluated using the 95% confidence interval (CI), log rank risk ratio (HR), and *p*-value.

ROC curves were produced and AUC values were computed using the R package pROC for ROC analysis and ggplot2 for visualization. An AUC value ranging from 0.5 to 0.7 signifies the presence of model success, while a value between 0.7 and 0.9 indicates a strong indication of model success. A value exceeding 0.9 denotes a robust indication of model success. Statistical analysis and visualization were conducted using R version 3.6.3.

### Analysis of genes differentially expressed with STAT3 in LGG

The present study employed Linked Omics to identify co-expressed genes of STAT3 in LGG. Subsequently, statistical analysis was conducted on the identified co-expressed genes, which were visualized in a heat map. Pearson’s test was utilized to assess the significant correlation of the co-expressed genes. A threshold of FDR <0.01 was deemed significant for gene expression, while a threshold of *p* < 0.05 was considered significant for gene correlation.

### Construction of a network of protein-protein interactions

The STRING database, which provides functional protein association networks, was utilized to predict protein-protein interactions (PPI) and construct the PPI network. Through this approach, the protein-protein interaction network of the STAT3 gene was predicted, revealing two primary protein clusters that exhibit close interaction. Subsequently, 50 genes were identified as direct interactors of STAT3 and subjected to KEGG enrichment analysis, resulting in the selection of 20 genes for the generation of a network diagram.

### KEGG enrichment analysis

The R software package “clusterprofiler” was utilized to conduct KEGG enrichment analysis and annotations plotting. The 50 genes that interact with STAT3 protein, as constructed by PPI network, underwent enrichment and analysis by KEGG. The gene sets exhibiting significant positive and negative correlation with PTPN2 expression, as identified by linkedomics, were intersected with the 50 genes with protein interaction, respectively. Subsequently, the two intersected gene sets were merged and subjected to KEGG enrichment analysis.

### Correlation analysis between the screened key genes and STAT3

The GEPIA2 online tool (cancer-pku.cn) was utilized to examine the expression correlation between the key genes of interest in the previously intersected genes and the STAT3 gene.

### Analysis of the expression and prognosis of identified pivotal genes

The present study utilized the online tool GEPIA2 GEPIA 2 (cancer-pku.cn) to examine the expression and prognostic implications of selected key genes of interest among the previously identified intersection genes.

### Cell culture

The LGG cancer cell lines U118 and U87, as well as the normal astrocyte HA1800 cell line, were procured from the American Type Culture Collection (ATCC) and maintained in endotoxin-free DMEM supplemented with 10% fetal bovine serum (FBS) (Gibco). The U87 cell line was cultured in MEM with 10% FBS. To prevent potential contamination, Penicillin-Streptomycin (C0222, Beyotime) and Plasmocin prophylactic (ant-mpp, InvivoGen) were added to all media in accordance with the manufacturer’s instructions.

### Transient overexpression of shNC and shSTAT3

The plasmids, pGV141-shNC and pGV141-shSTAT3, were procured and constructed from Genechem Co. Ltd. (Shanghai, China). The experimental procedure involved seeding 4 × 10^5^ cells onto a 6-well plate and allowing them to adhere to 70% confluence. Subsequently, 200 μL of transfection complex, comprising 1 μg plasmid and 1 μL X-tremeGENE HP DNA Transfection Reagent (Roche, 06366236001), was added to each well. The expressions were validated by RT-qPCR after 24–48 h of transfection. The target sequences of shSTAT3 and shNC were listed in Supplementary Table S1.

### Q-PCR analysis

The RNA extraction process involved the use of RNA-easy Isolation Reagent in accordance with the manufacturer’s instructions. The extracted RNA was then subjected to reverse transcription to cDNA using HiScript^®^ III RT SuperMix (Vazyme, R323-01) and subsequently analyzed through qRT-PCR using ChamQ SYBR qPCR Master Mix (Low ROX premix) (Vazyme, Q331-02). The qPCR assays were conducted using an ABI 7500 Fast Real-Time PCR system (Applied Biosystem, United States) and the primers utilized are presented in Supplementary Table S2. Each sample was analyzed in triplicate in this study. The relative gene expressions were determined using the 2^−ΔΔCt^, with GAPDH serving as the endogenous control.

### Statistical analysis

Data were analyzed using SPSS. Results were expressed as means ± SD. Differences between treatment regimens were analyzed by one way ANOVA. *p* < 0.05 was considered statistical significance.

## Results

### Transcriptional levels of STATs in patients with brain and CCNS cancers

**FIGURE 1:**
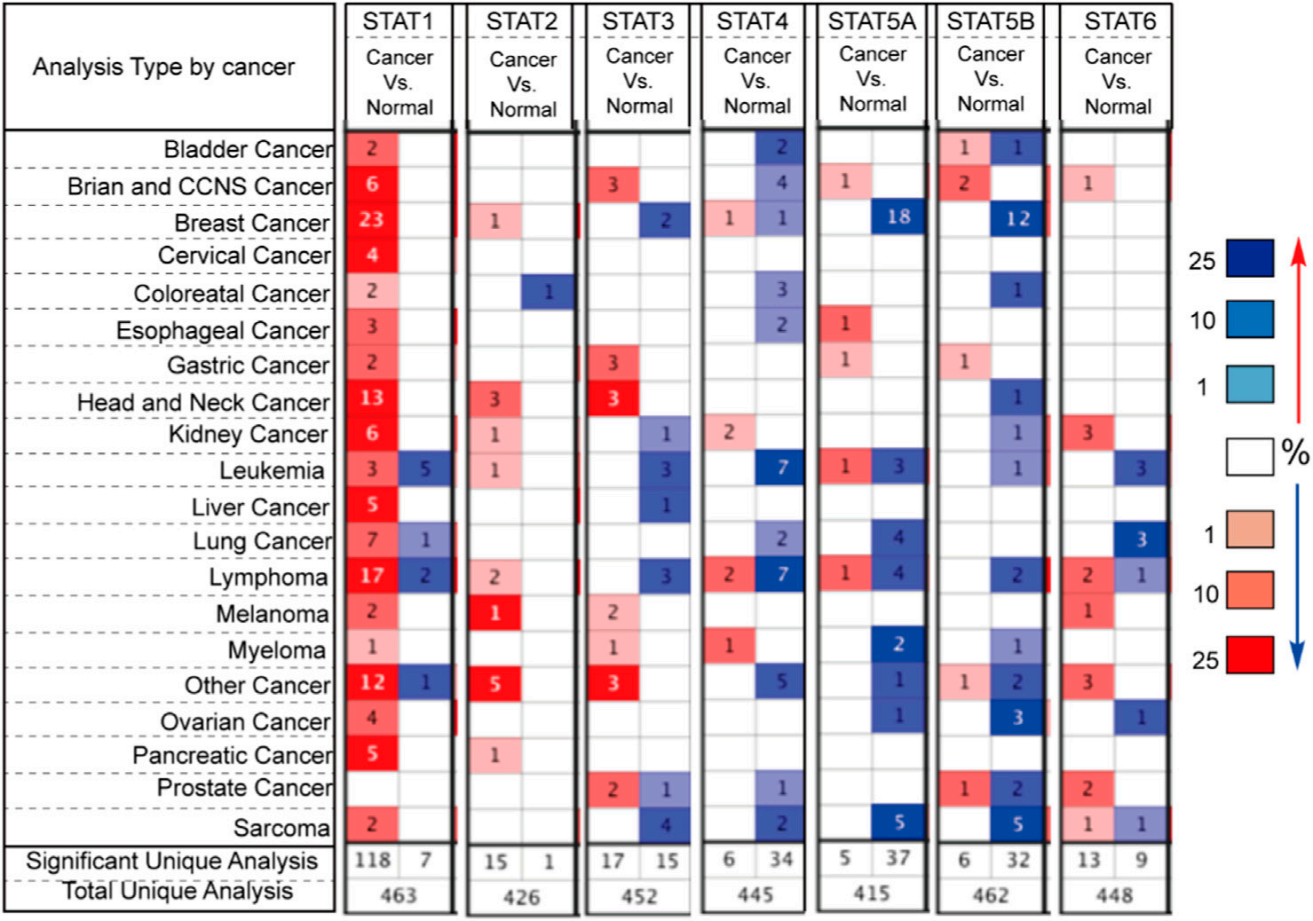
The Transcription Levels of STATs in Different Types of Cancers in the Oncomine database. Red and blue respectively represent cancer and normal tissues. The darker color indicates higher expression of gene. Samples from all cancers with target gene expression information used to total unique analysis. Samples from all cancers with target gene differential expression information used to significant unique analysis. The number in the square represents the number of datasets that met our screening requirements (*p* < 0.05).

Seven STATs have been identified in mammalian cells. In this study, we utilized Oncomine databases to compare the expression levels of STATs in cancer and normal samples (Figure 1). Our findings indicate that the mRNA expression level of STAT1 is significantly upregulated in all types of cancers, with the exception of prostate cancer. The results in TCGA also support the conclusion that STAT1 is overexpressed in various cancers (Table 1). Subsequently, we compared the mRNA expression level of STAT2 in cancer and normal samples. The results indicate that STAT2 is also overexpressed in many types of cancers. Further analysis showed that there was no significant difference in STAT2 expression in LGG compared with normal tissues (Figure 1). Different from STAT2, STAT1, STAT3, STAT5A, STAT5B, and STAT6 were found to be expressed at higher levels in Brian and CCNS cancer compared to normal samples. The expression of STAT4 was lower in Brian and CCNS cancer compared to normal samples (Figure 1). Considering the small sample data of Oncomine databases, we also analyzed the differential mRNA expression levels of STATs in TCGA. The results confirmed that the expression of STAT1, STAT3, and STAT5A were higher in LGG compared to normal samples (Table 1). However, the expression of STAT4 and STAT6 were low in LGG compared to normal samples (Table 1). Subsequently, we evaluated the relationship of STATs expression and cancer malignant progression by analyzing survival and prognosis. As shown in Table 1, the overexpression of STAT family members is correlated to the malignant progression of some cancers.

**TABLE 1:** The distribution of STATs in different cancers assessed by expression and hazard.

Cancers	Overexpression members^a^	Survival analysis^b^	Prognostic analysis^c^
Adrenocortical carcinoma	No^d^	STAT1	STAT2, STAT3, STAT5A, STAT5B
Breast cancer	STAT1	No^d^	STAT5A, STAT5B
Cervical cancer	STAT1	No^d^	STAT1, STAT5B
Cholangiocarcinoma	No^d^	No^d^	STAT1, STAT2, STAT5A, STAT5B, STAT6
Colorectal cancer	STAT1	No^d^	STAT1, STAT2, STAT6
Lymphoma	STAT1, STAT5A	No^d^	No^d^
Esophageal cancer	STAT1	No^d^	STAT1
Glioma	STAT1, STAT3, STAT5A	STAT1	STAT1, STAT3
Head and Neck cancer	STAT1, STAT2	No^d^	STAT2
Kidney cancer	STAT4	STAT1, STAT2	STAT4
Leukemia	STAT2, STAT4, STAT6	STAT4, STAT6	STAT2, STAT4, STAT6
LGG	STAT1, STAT3, STAT5A	STAT1, STAT2, STAT3, STAT4, STAT5A, STAT6	STAT3
Liver cancer	STAT1	No^d^	No^d^
Lung cancer	STAT1	No^d^	STAT5A, STAT5B
Ovarian cancer	STAT1	STAT5A	STAT1, STAT2, STAT5A, STAT5B, STAT6
Pancreatic cancer	STAT1, STAT3, STAT5A, STAT6	No^d^	STAT1, STAT5A
Prostate cancer	No^d^	STAT2, STAT4	STAT5B
Cutaneous Melanoma	STAT1	No^d^	No^d^
Gastric cancer	STAT1	No^d^	STAT1
Testicular cancer	STAT1	No^d^	STAT1, STAT4, STAT5B
Thyroid cancer	STAT1	No^d^	No^d^
Thymoma	STAT1, STAT2	No^d^	No^d^
Uterine cancer	STAT1	No^d^	STAT5A, STAT5B, STAT6
Melanoma	No^d^	STAT1, STAT3, STAT4, STAT5B, STAT6	No^d^

^a^ The differential expression of STATs, between tumor tissues and normal tissues was analyzed by GEPIA, two in TCGA, and GTEx. List genes are overexpressed in tumor tissues. Data were statically analyzed using one way ANOVA, *p*-value <0.01.

^b^ The association between patient survival (OS, and DFS) and STATs, gene expression was analyzed by GEPIA, two in TCGA., the high expression of list genes leads to shorter survival, where HR (high) > 1.0, Logrank *p* < 0.05.

^c^ The relationship of STATs, expression and the cancer malignant progression was analyzed data in TCGA, or XENA, by ROC, curves. List genes (AUC, value >0.9) are positively correlated to the malignant progression of cancers.

^d^ No genes distribute in this type of cancer.

### Correlation between the mRNA levels of STATs and the clinicopathological parameters of patients with LGG

**FIGURE 2:**
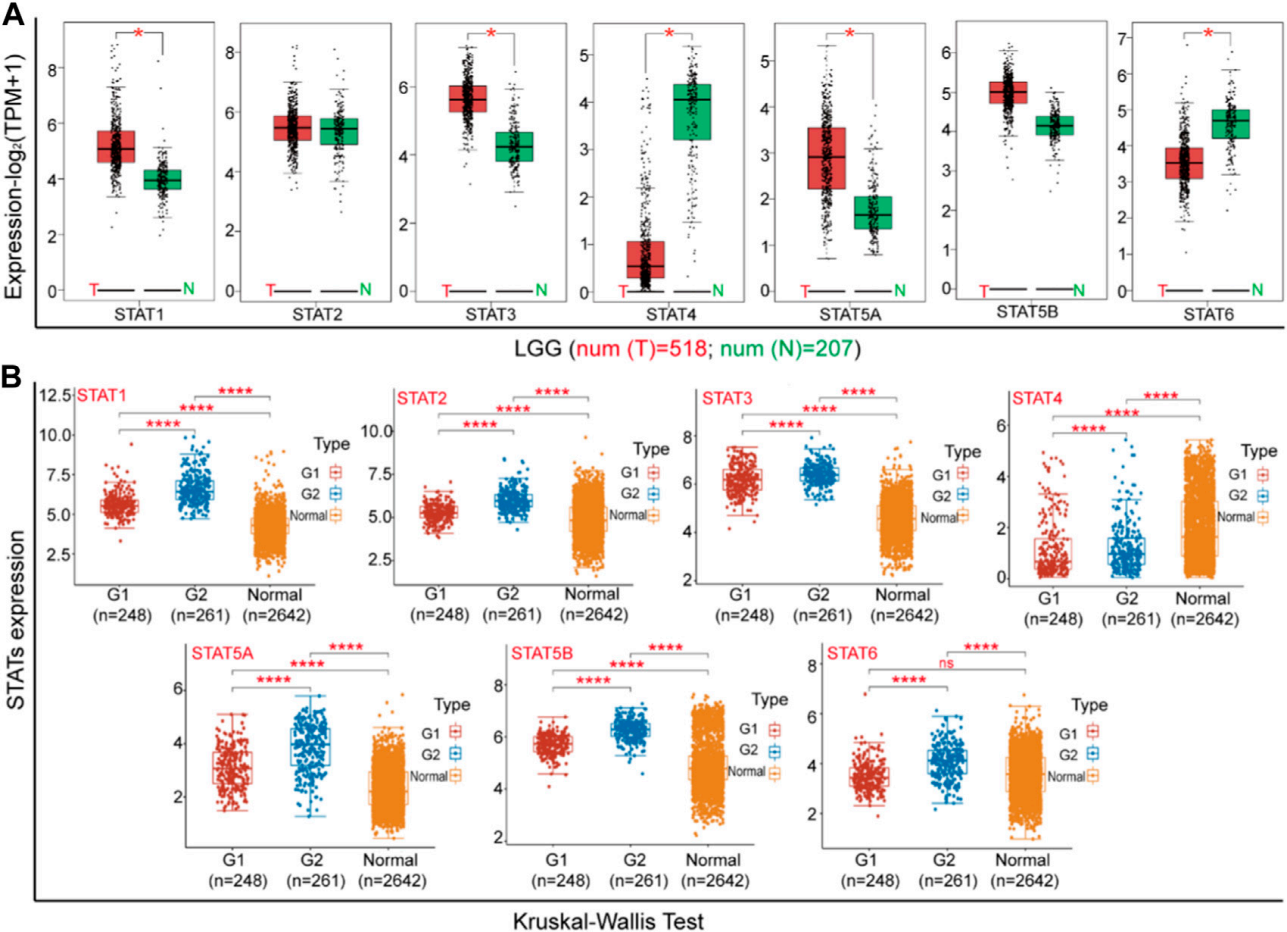
The expression of STATs in Brian Lower Grade Glioma (LGG). **(A)** The differential expression of STATs between cancerous tissues and adjacent tissues (GEPIA). **(B)** The expression of STATs at different stage (xiantao love).

Using the Gene Expression Profiling Interactive Analysis (GEPIA) dataset and the online analytical software Xiantao Love, we conducted a comparison of mRNA expression levels of STATs factors between Brian Low-grade glioma (LGG) tissue and adjacent tissues of the TCGA project. Our findings revealed that the expression levels of STAT1, STAT3, STAT5A, and STAT6 were significantly higher in LGG tissue compared to normal tissues, while the expression level of STAT4 was lower in the former than in the latter (Figure 2A). Furthermore, we proceeded to analyze the expression of STATs with respect to tumor stage for LGG (Figure 2B). The present study revealed that the expression levels of STAT1, STAT2, STAT5A, and STAT5B were significantly elevated in LGG tissue compared to normal tissues, with higher expression levels observed in G2 stage relative to G1 stage. Additionally, the expression of STAT6 was found to be upregulated in LGG tissue at G2 stage compared to normal tissues. Conversely, the expression of STAT4 was observed to be downregulated in LGG tissue compared to normal tissues.

**FIGURE 3:**
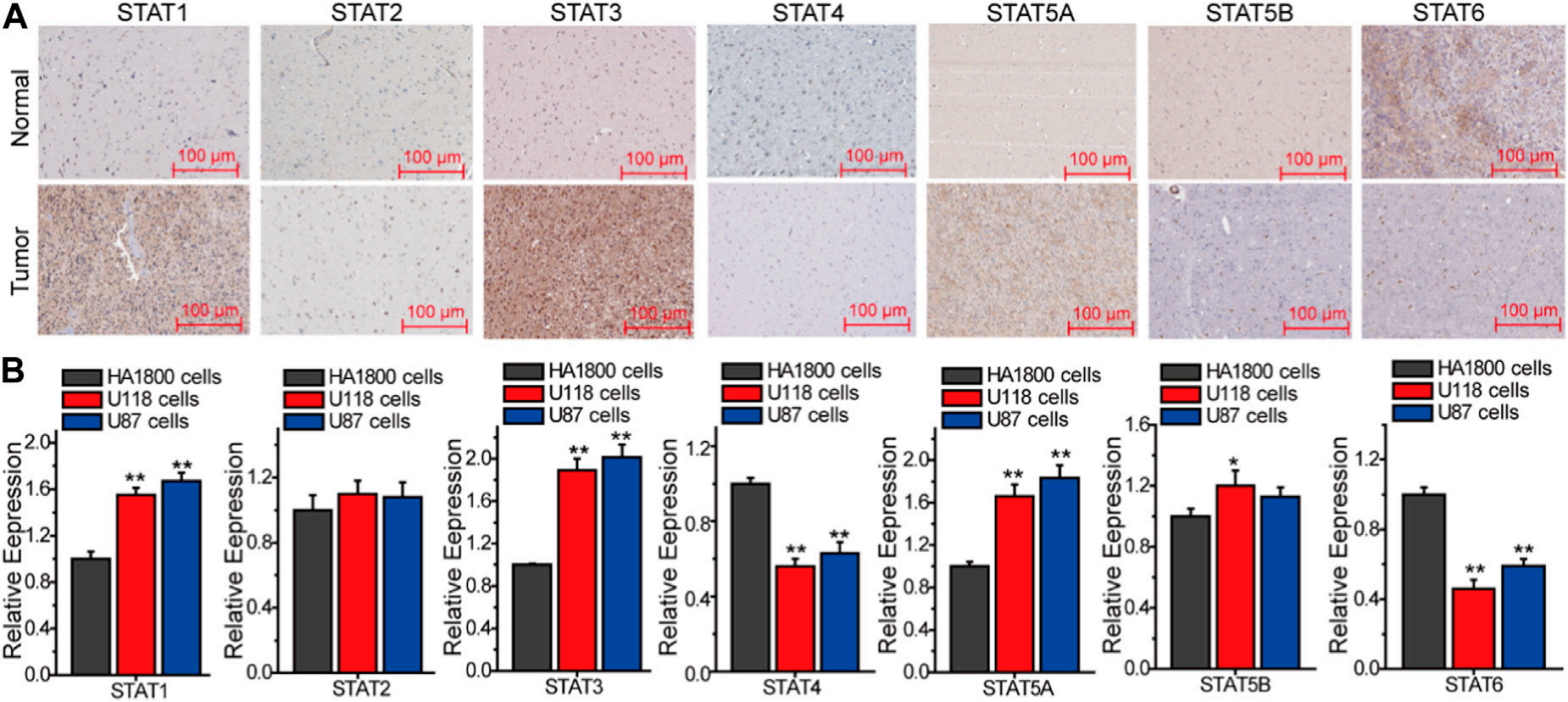
The expression of STATs in Gliomas. **(A)** The differential expression of STATs between tumor tissues and normal tissues (PROTEIN ATLAS). **(B)** The expression of STATs in HA 1800, U118 and U87 cells. The results from three independent experiments were statistically analyzed using one way ANOVA: ^*^ *p* < 0.05, ^**^ *p* < 0.01 compared with HA1800 cells.

In order to provide additional evidence for the association between STATs and the malignant advancement of LGG, an analysis was conducted to compare the expression levels of STATs in tumor tissues and normal tissues (as depicted in Figure 3). The immunohistochemical data pertaining to STATs expression in both brain glioma tissues and normal brain tissues were sourced from the human protein atlas database. Figure 3A illustrates that the expression levels of STAT1, STAT3, and STAT5A were elevated in Brian glioma tissues compared to normal Brian tissues, while the expression level of STAT4 was lower in the former than in the latter. Conversely, there were no significant differences in the expressions of STAT2 and STAT5B between Brian glioma tissues and normal Brian tissues (Figure 3A). Following this, we conducted Q-PCR to assess the expression of STATs genes in human normal astrocytes HA 1800, glioma cell lines U118 and U87 (Figure 3B). The findings indicate that the levels of STAT1, STAT3, and STAT5A were elevated in glioma cells compared to normal astrocytes, while the expression of STAT4 and STAT6 was reduced in the former relative to the latter. Conversely, there was no significant difference in the expression of STAT2 and STAT5B between these cell types.

### Association of the mRNA expression of STATs with the improved prognosis of patients with LGG

**FIGURE 4:**
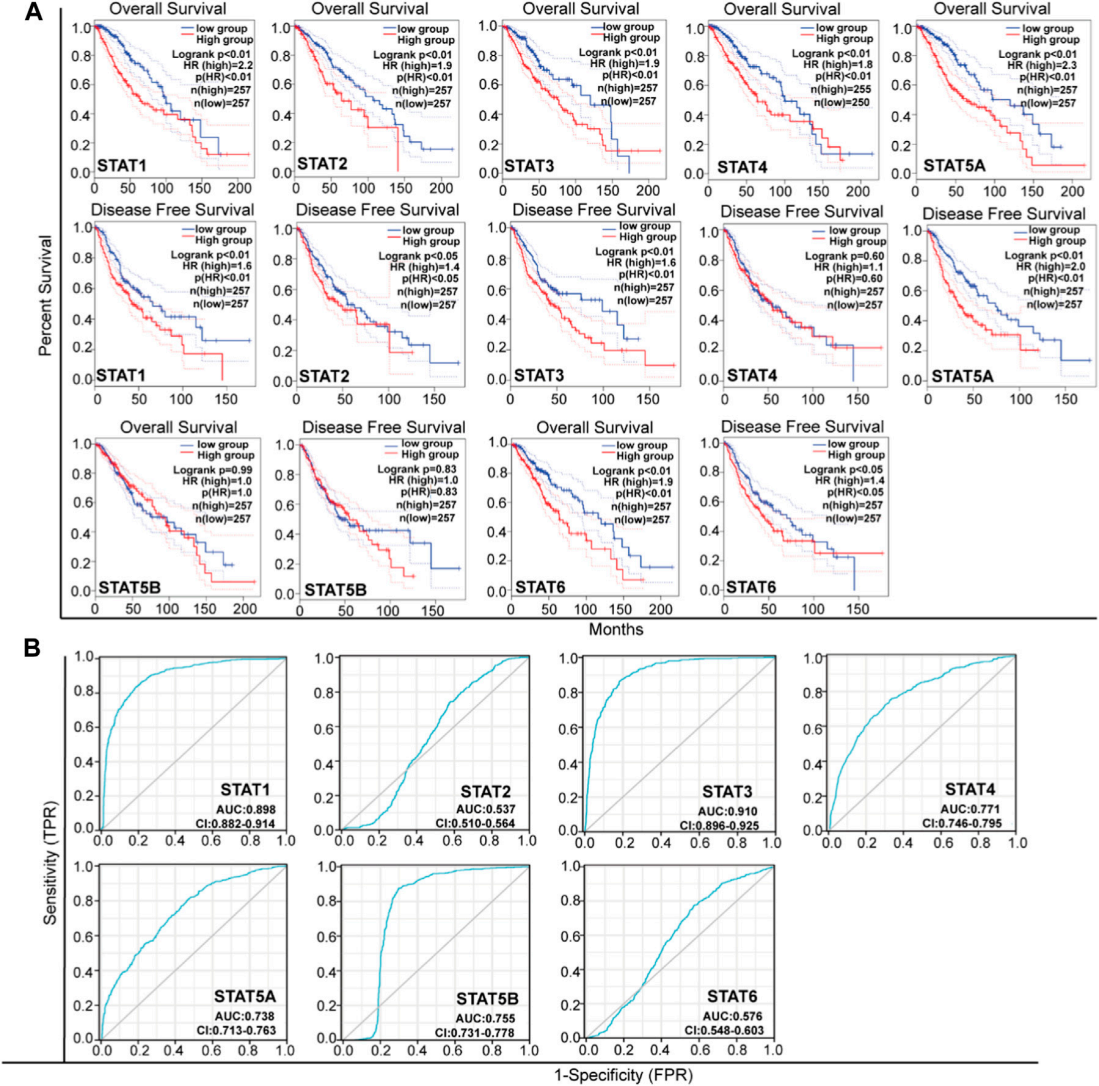
The Prognostic Value of mRNA level of STATs in LGG. **(A)** Analyze the association between patient survival and STATs expression (GEPIA). **(B)** ROC curves for the relationship of STATs expression and the poor prognosis of LGG. An AUC value ranging from 0.5 to 0.7 signifies the presence of model success, while a value between 0.7 and 0.9 indicates a strong indication of model success. A value exceeding 0.9 denotes a robust indication of model success.

In this study, we conducted an in-depth investigation into the crucial efficacy of STATs in relation to the malignant progression of LGG. To achieve this, we utilized GEPIA tools to analyze the correlation between the mRNA levels of STATs and the survival rates of LGG patients, using data obtained from the TCGA project (Figure 4A; Table 1). Our log rank test analyses revealed that elevated levels of STAT1, STAT2, STAT3, STAT5A, and STAT6 mRNA were significantly linked to both overall survival (OS) and disease-free survival (DFS) in all LGG patients. Furthermore, an investigation was conducted to examine the impact of STATs expression on the malignant progression of LGG through the utilization of receiver operating characteristic (ROC) curves (Figure 4B). The ROC curves of STAT1, STAT4, STAT5A, and STAT5B exhibited area under the curve (AUC) values ranging from 0.7 to 0.9, indicating that the expressions of these STATs may be associated with the progression of LGG. Additionally, the AUC value of the ROC curve of STAT3 was 0.91, signifying a positive correlation between STAT3 expression and the malignant progression of LGG (Figure 4B; Table 1).

### Correlation between the expression of STAT3 and the malignant progression of LGG

The preceding analysis demonstrated a positive correlation between the expression level of STAT3 and the malignant progression of LGG. Consequently, we investigated the association between the mRNA expression of STAT3 and the hazard ratio of patients with LGG, utilizing the Xiantao love online software (Table 2). The findings indicated that the hazard ratio of patients with LGG was positively associated with the stage of LGG, the age of the patient, and the mRNA expression level of STAT3. The univariate analysis of the hazard ratio confidence interval (CI) of STAT3 was 2.41, which was significantly higher than the reference (Table 2).

**TABLE 2:** The effect of different indicators on the hazard ratio of LGG.

Characteristics	Total (N)	Univariate analysis		Multivariate analysis	
		Hazard ratio (95% CI)	*p*-Value	Hazard ratio (95% CI)	*p*-Value
WHO grade	466				
G2	223	References			
G3	243	3.06 (2.05–4.57)	**<0.001**	2.43 (1.54–3.81)	**<0.001**
Primary therapy outcome	457				
PD	110	References			
SD	146	0.44 (0.29–0.66)	**<0.001**	0.34 (0.21–0.55)	**<0.001**
PR	64	0.18 (0.08–0.40)	**<0.001**	0.15 (0.05–0.42)	**<0.001**
CR	137	0.12 (0.06–0.27)	**<0.001**	0.13 (0.06–0.29)	**<0.001**
STAT3	527	2.41 (1.68–3.45)	**<0.001**	1.20 (0.75–1.94)	0.451
Age	527				
<=40	264	References			
>40	263	2.89 (2.00–4.16)	**<0.001**	3.11 (1.99–4.85)	**<0.001**
Histological type	527				
Astrocytoma	195	References			
Oligoastrocytoma	134	0.66 (0.42–1.04)	0.071	1.51 (0.89–2.56)	0.126
Oligodendroglioma	198	0.58 (0.39–0.85)	**0.005**	0.54 (0.33–0.89)	**0.016**

The bold values indicate statistical significance.

### The regulation of STAT3 in the malignant progression of LGG

**FIGURE 5:**
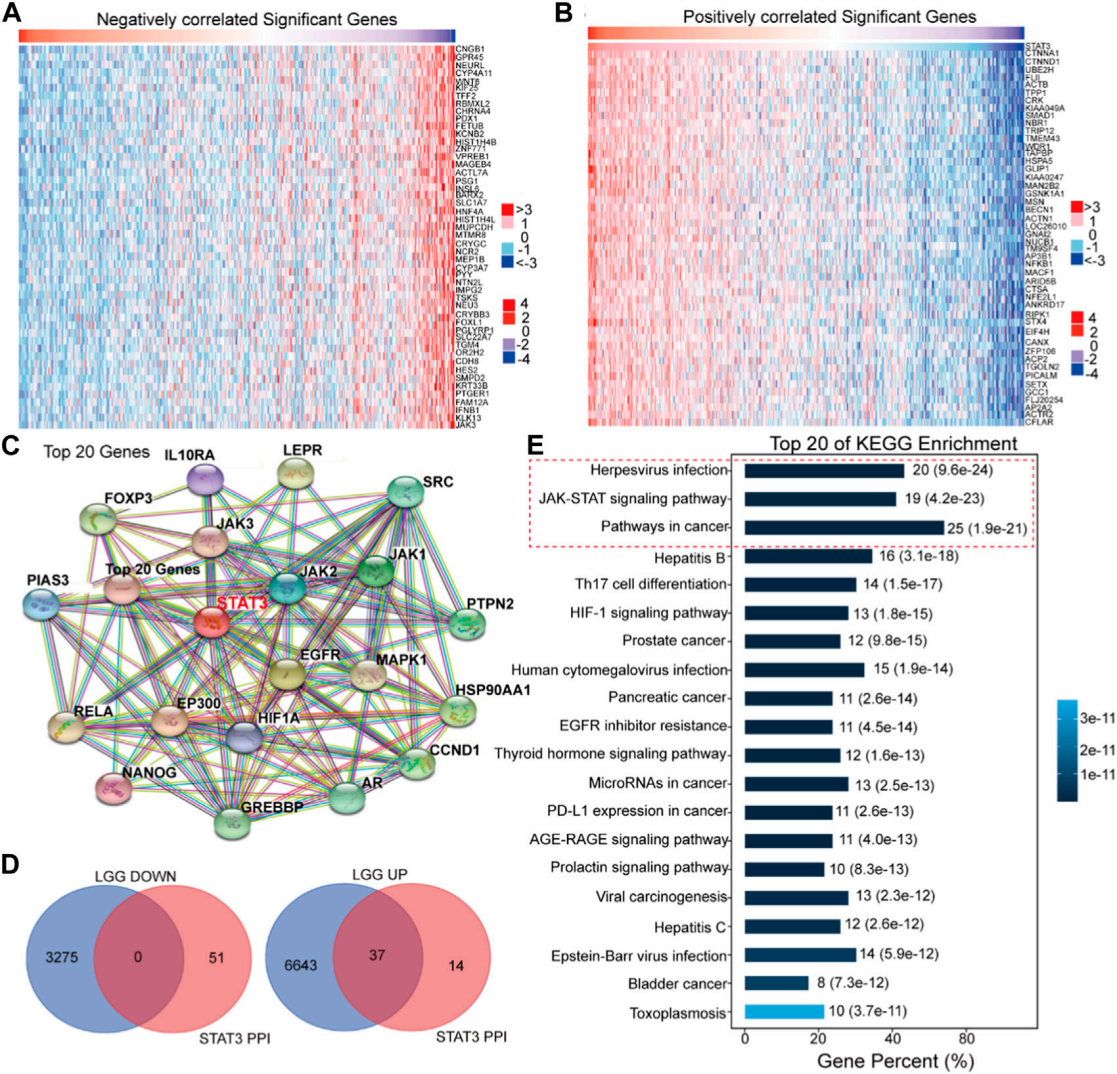
Bioinformatics analysis of the regulation of STAT3 in the malignant progression of LGG. **(A)** The negatively correlated significant genes of STAT3 in LGG (EMTome). **(B)** The positively correlated significant genes of STAT3 in LGG (EMTome). **(C)** The PPI analysis of STAT3 (STRING). **(D)** Online prediction the common genes in STAT3 PPI network and STAT3 correlated genes (BioVenn). **(E)** KEGG enrichment of the common genes (Omicshare).

In order to elucidate the regulatory mechanisms of STAT3 in the malignant progression of LGG, a systematic analysis was conducted on the roles of STAT3 in LGG patients utilizing the EMTome online software (Figures 5A,B). The results of the analysis revealed that 3,275 genes exhibited a negative correlation with the expression of STAT3 in LGG, while 6,680 genes exhibited a positive correlation with the expression of STAT3 in LGG, as depicted in the heat map (Figures 5A,B). Furthermore, a protein-protein interaction (PPI) network was constructed utilizing STAT3 as the central node, as illustrated in Figure 5C. In Figure 5D, it was observed that 37 genes were present in both the PPI network-related genes of STAT3 and the positively correlated genes. The Omicsmart online platform was utilized to perform KEGG pathway enrichment analysis on these 37 genes, revealing that the primary pathways associated with them were Herpesvirus infection, JAK-STAT signaling pathway, and pathways in cancer (Figure 5E). Based on the outcomes of the KEGG enrichment analysis, a subset of 15 genes that were involved in the aforementioned pathways were selected.

**FIGURE 6:**
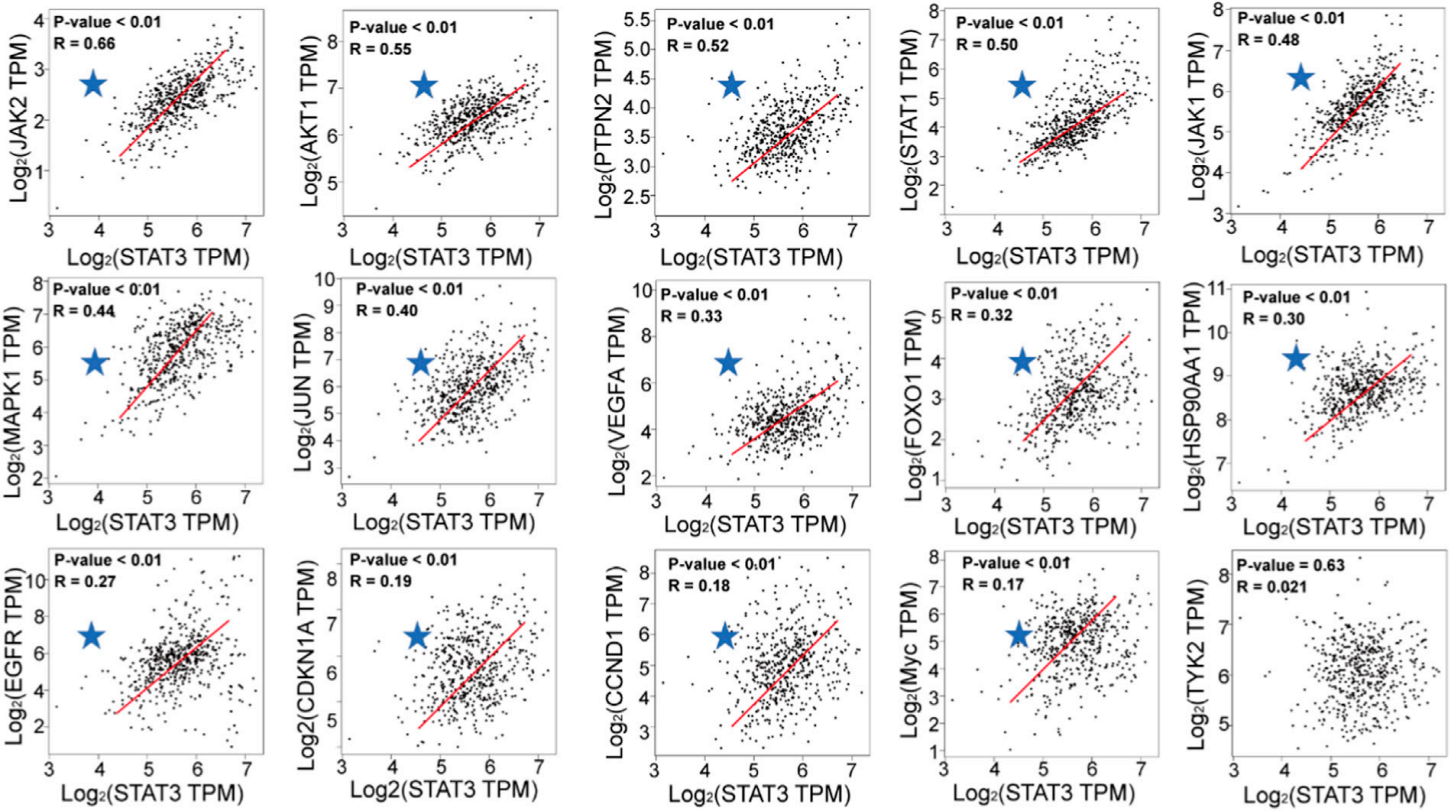
Correlation analysis of STAT3 expression and key genes expression in LGG (GEPIA).

In order to elucidate the correlation between STAT3 and a chosen set of 15 genes, we conducted an analysis of their respective expression patterns with respect to one another. Following correlation analysis, a positive correlation was observed between the expression of STAT3 and that of Janus kinase 2 (JAK2), AKT1, protein tyrosine phosphatase non-receptor type 2 (PTPN2), STAT1, Janus kinase 2 (JAK1), mitogen-activated protein kinase 1 (MAPK1), JUN, vascular endothelial growth factor A (VEGFA), forkhead box O1 (FOXO1), heat shock protein 90 alpha family class A member 1 (HSP90AA1), epidermal growth factor receptor (EGFR), cyclin dependent kinase inhibitor 1A (CDKN1A), cyclin D1 (CCND1), and Myc (Figure 6).

### The regulation of STAT3 in LGG progression involves the participation of STAT1, FOXO1, and MYC

**FIGURE 7:**
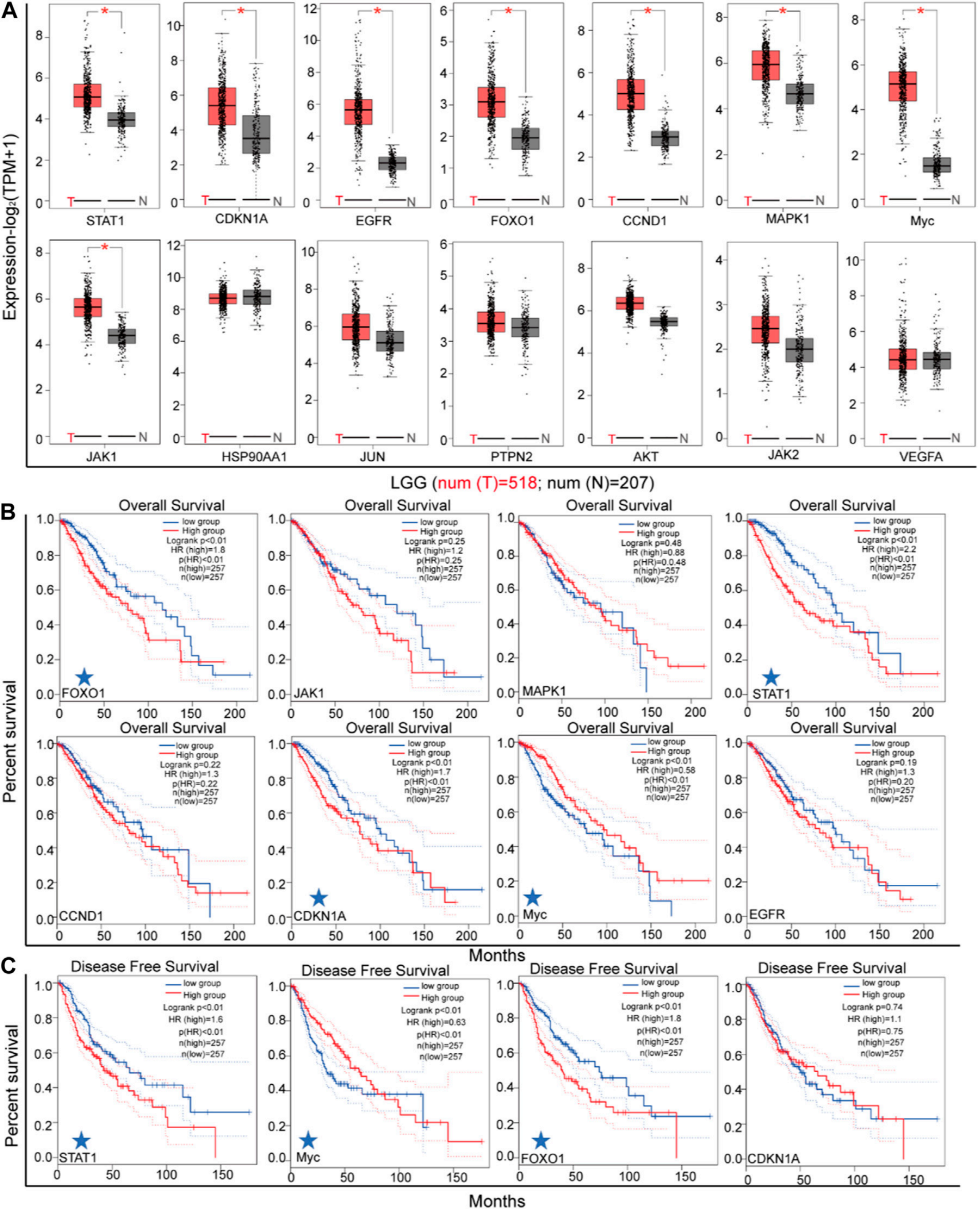
The expression of key genes in LGG. **(A)** The differential expression of key genes between LGG tissues and adjacent tissues (GEPIA). **(B)** The association between patient overall survival and genes expression (GEPIA). **(C)** The association between disease free survival and genes expression (GEPIA).

In order to identify the genes most likely to be involved in the regulation of STAT3 in LGG malignant progression, an analysis was conducted on the differential expressions of 14 genes in LGG tissues and normal adjacent tissues (as depicted in Figure 7A). The mRNA expressions of STAT1, CDKN1A, EGFR, FOXO1, CCND1, MAPK1, Myc, and JAK1 were found to be higher in LGG tissues than in normal adjacent tissues. Subsequently, the correlation between the mRNA levels of these eight genes and the survival of patients with LGG was analyzed (as shown in Figure 7A). The results of the log rank test analyses indicate a significant association between increased levels of STAT1, FOXO1, and Myc and the overall survival (OS) and disease-free survival (DFS) of LGG, as depicted in Figures 7B,C.

**FIGURE 8:**
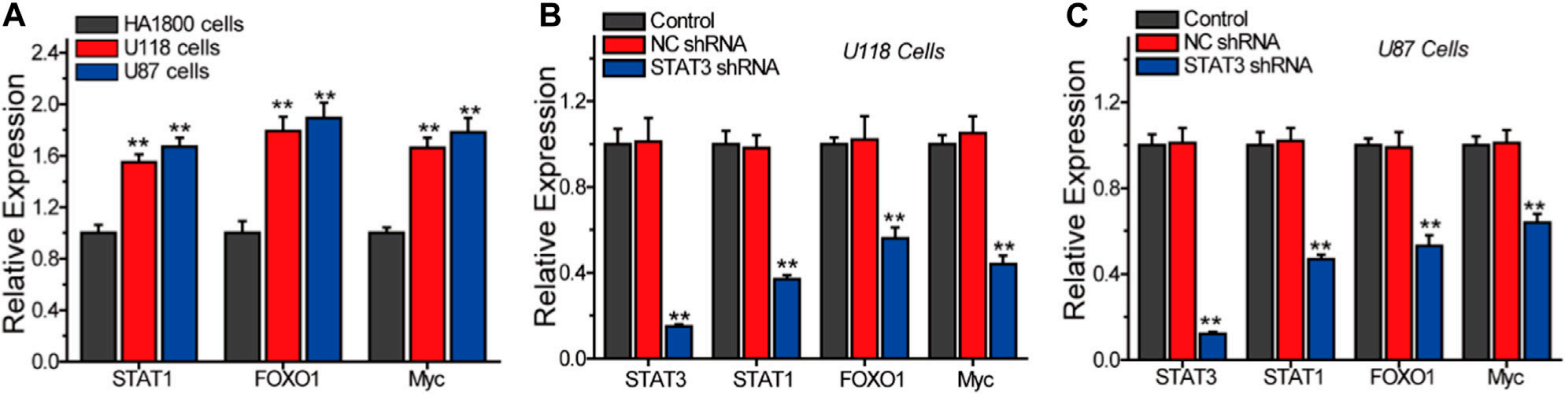
Q-PCR analyze the relative expression of STAT3, STAT1, FOXO1 and Myc in different cells. **(A)** The expression of STAT1, FOXO1 and Myc in HA 1800, U118 and U87 cells. The effects of STAT3 on the expression of STAT1, FOXO1 and Myc in U118 cells **(B)** and U87 cells **(C)**.

In order to further validate the involvement of STAT1, FOXO1, and Myc as key genes in the STAT3-regulated malignant progression of LGG, we conducted Q-PCR analysis to assess the expression of these genes under different conditions, as illustrated in Figure 8. Our findings demonstrate a significant increase in the expression of STAT1, FOXO1, and Myc in glioma cells U118 and U87, as compared to normal astrocytes HA 1800 (Figure 8A). The inhibition of STAT3 expression in U118 and U87 glioma cells resulted in a significant reduction in the expressions of STAT1, FOXO1, and MYC (Figures 8B,C). These findings indicate that STAT1, FOXO1, and Myc are genes that play a role in the STAT3-regulated signaling pathway.

## Discussion

LGGs, being the most prevalent brain tumors in children, are currently classified by the World Health Organisation as grades I and II (Ryall et al., 2020). The diagnosis and treatment of patients with LGGs pose a challenge due to the heterogeneity in their clinical behavior (Wang and Mehta, 2019; Ryall et al., 2020). Presently, the primary strategies for glioma treatment include surgery, chemotherapy, and/or radiotherapy. Nevertheless, the adverse effects and drug resistance associated with chemotherapy and radiotherapy often lead to treatment failures (Wang and Mehta, 2019). Hence, the identification of biomarkers and therapeutic targets for gliomas is of paramount importance. The dysregulation of STAT family members has been documented in numerous cancers, indicating their involvement in cancer progression by integrating signals from diverse signaling pathways (Loh et al., 2019; Verhoeven et al., 2020). Therefore, investigating the association between STAT expression and the malignant progression of gliomas is imperative to identify potential targets for diagnosis and treatment. In this study, we examine the correlation between STAT expression and the progression of LGG.

Each member of the STAT family performs distinct functions in signal transduction and facilitates cellular responses to various cytokines (Loh et al., 2019). Our research has revealed that STAT1 is frequently upregulated in numerous cancers and is positively correlated with the malignant progression of LGG (Yang et al., 2020). While the majority of evidence suggests that STAT1 functions as a tumor suppressor in cancer cells, some studies have indicated that under specific conditions, STAT1 may exert tumor promoter effects (Yang et al., 2020). Hence, it is plausible that STAT1 may act as a tumor promoter in gliomas. Conversely, the mRNA expression of STAT2 does not appear to be associated with the malignant progression of LGG. The constitutive activation of STAT3 is essential for the carcinogenesis of head and neck cancer, and there is evidence to suggest that STAT3 is involved in the transition from LGG to high grade glioma (Doucette et al., 2012; Lee et al., 2019). Our research indicates that STAT3 is overexpressed in LGG and contributes to the malignant progression of gliomas. Analogous to STAT3, dysregulated STAT5 signaling has been linked to elevated cell proliferation, survival, and metastasis in various cancers (Halim et al., 2020). STAT5 comprises two subtypes, namely, STAT5A and STAT5B (Zhang and Liu, 2017). Our investigation revealed that the expression and biological activity of STAT5A and STAT5B differed. Specifically, STAT5A was found to be upregulated in LGG tissues and exhibited a positive correlation with the malignant advancement of gliomas. The correlation between STAT5B and the progression of LGG was found to be insignificant. Our study has emphasized the predominant role of STAT5A in gliomas. The expression and function of STAT4 and STAT6 in gliomas is a topic of interest. Upon analysis, it was determined that the mRNA expression of STAT4 and STAT6 was comparatively lower in glioma tissues as opposed to adjacent tissues. However, the results of survival analysis indicate that the overexpression of STAT4 and STAT6 promote the malignant progression of LGG. STAT4 is known to localize to the cytoplasm and bind to the membrane following phosphorylation. The activation of STAT4 is deemed critical for the promotion of cellular-mediated immune response through multiple signaling pathways (Maurer et al., 2019). STAT6 has been linked to tumorigenesis, immunosuppression, proliferation, metastasis, and unfavorable prognosis (Huang et al., 2020). As pro-cancer factors, although the expression levels of STAT4 and STAT6 are relative low, they may play an important role in the malignant transformation of LGG and the occurrence of drug resistance. As we know, LGG often progresses to secondary malignant transformation (Lucke-Wold et al., 2024). In this malignant transformation, the expression levels of STAT4 and STAT6 may be elevated, which further promotes this malignant transformation. Meanwhile, LGG easily develops resistance to radiotherapy, chemotherapy, and immunotherapy (Hayes et al., 2018). The expression levels of STAT4 and STAT6 may also increase to promote the process of LGG developing drug resistance. Therefore, it is worth paying attention to evaluate the expression levels of STAT4 and STAT6 during the malignant transformation of LGG. Among the STAT family members, STAT3 exhibited the strongest effect on the malignancy of gliomas, and thus, was selected for further analysis.

Upon analysis, it was determined that 6,680 genes exhibited a positive correlation with the expression of STAT3 in LGG. Among these genes, 37 were found to be involved in the STAT3 PPI network. Through systematic analysis, it was discovered that STAT1, FOXO1, and MYC were pivotal factors in the regulation of STAT3 during the progression of LGG. The expression levels of STAT1, FOXO1, and MYC were observed to be higher in gliomas compared to normal astrocytes and exhibited a positive correlation with the expression of STAT3. The prevailing consensus is that the transcription factor FOXO1 impedes cancer progression through the activation of cell apoptosis and inhibition of cell metastasis (Kim et al., 2018). Some studies have shown that FOXO1 plays a pivotal role as a tumor suppressor in lung cancer (Ebrahimnezhad et al., 2023). However, many researches suggest that FOXO1 promotes the malignant progression of leukemia (Liu et al., 2019). Tomiyasu *et al* also regard that FOXO1 supports the malignant proliferation of breast cancer cells that and coloreatal cancer cells by promoting p53 degradation (Tomiyasu et al., 2024). Hence, the biological role of FOXO1 in cancer progression is contingent upon the tumor type (Kim et al., 2018; Liu et al., 2019; Ebrahimnezhad et al., 2023; Tomiyasu et al., 2024). Specifically, the mRNA expression level of FOXO1 exhibited a positive correlation with the progression of LGG, and the repression of FOXO1 was induced by the silencing of STAT3. The induction of FOXO1 by STAT3 has been found to promote the malignant progression of LGG. The deregulation of the oncogene MYC is a common occurrence in many cancers and is often associated with poor prognosis and unfavorable survival (Chen et al., 2018). Our research has revealed that MYC is overexpressed in LGG tissues and that repression of STAT3 leads to a decrease in Myc expression. These findings suggest that STAT3 plays a crucial role in the malignant progression of LGG by regulating the expression of STAT1, FOXO1, and MYC.

## Conclusion

In brief, the present study examined the impact of STAT family members on the malignant advancement of LGG. Our findings indicate that STAT1, STAT3, and STAT5A were upregulated and facilitated the progression of LGG. Conversely, STAT4 and STAT6 were found to promote glioma progression by inhibiting the antitumor immune response. The effects of STAT2 and STAT5B on the malignant progression of LGG were deemed insignificant. Among these members, STAT3 was identified as the most significant factor in regulating the progression of LGG by facilitating the expression of STAT1, FOXO1, and MYC. STAT3, STAT1, FOXO1, and MYC, play a crucial role in driving the malignant progression of LGG. Therefore, targeting STAT3 represents a promising therapeutic strategy for patients afflicted with gliomas.

## Data Availability

The datasets presented in this study can be found in online repositories. The names of the repository/repositories and accession number(s) can be found below: https://doi.org/10.6084/m9.figshare.26027179.v1.
