# Supplementary material for: STAT3 drives the malignant progression of low-grade gliomas through modulating the expression of STAT1, FOXO1, and MYC
Source: Front Mol Biosci. 2024 Jun 14;11:1419072. doi: 10.3389/fmolb.2024.1419072 (PMC11211654; doi:10.3389/fmolb.2024.1419072)
Supplement: Supplementary file 1 [file Table1.doc]

**Supplemental Materials for**

**STAT3 drives the malignant progression of low-grade gliomas through modulating the expression of STAT1, FOXO1, and MYC**

Yan Li^1#^, Fanjing Jiang^1#^, Suhua Zhu^1^, Hongwei Jia^1^*, Changwei Li^1^*

^1^ Department of pharmacy, The Affiliated Xuzhou Municipal Hospital of Xuzhou Medical University, Xuzhou First People's Hospital, Xuzhou 22116, China

# Yan Li and Fanjing Jiang contributed equally to this article

* Corresponding authors: Changwei Li, Email: 15862278909@163.com; #269 University Road, Tongshan District, Xuzhou 221116, Jiangsu, China. Tel: +86-15862278909; Hongwei Jia, 15162143093@163.com

**Table S1. Sequences for shRNA.**

| Name | Sequence |
| --- | --- |
| NC shRNA | CCGGCATTCTCCGAACGTGTCACGTCTCGAGACGTGACACGTTCGGAGAATTTTTG |
| STAT3 shRNA1 | CCGGCGCGTCCAGTTCACTACTAAAGCTCGAGCTTTAGTAGTGAACTGGACGCTTTTTG |
| STAT3 shRNA2 | CCGGCGGTACATCATGGGCTTTATCACTCGAGTGATAAAGCCCATGATGTACCTTTTTG |
| STAT3 shRNA3 | CCGGCGCAACAGATTGCCTGCATTGGCTCGAGCCAATGCAGGCAATCTGTTGCTTTTTG |

**Table S2.** Primer sequences for genes in Q-PCR.

| **Name** | **Sense (5’-3’)** | **Antisense (5’-3’)** |
| --- | --- | --- |
| STAT1 | AGATTTAATCAGGCTCAGTCG | GGCTCTTGATTTCATGCTCT |
| STAT2 | AGCCAGTTCTCGAAACACC | ACCAGCTTCTCCATCATAGCC |
| STAT3 | AGGTTGCTGGTCAAATTCCC | TCTGAGAGCTGCAACGTCCC |
| STAT4 | GCCAGAACTAAACTATCAGGT | TGGCTTTGACATTAGTTCCAC |
| STAT5A | AATGAGAACACCCGCAACGAG | GGTCAGCACGCTTGATCCTCT |
| STAT5B | GCCCACTTCAGGAATATGTCCC | CCAGCTCATTTCCACCAACACT |
| STAT6 | TGAAGTCCTGAGAACCCTCGT | CAAGCCCAACAGGAATCGAAC |
| FOXO1 | TCACCCAGCCCAAACTACCAA | CTTCAAGAGTCCAGGCGCACA |
| MYC | ATACATCCTGTCCGTCCAAGC | TACGCACAAGAGTTCCGTAGC |
| GAPDH | GAAACTGTGGCGTGATGGC | CACCACTGACACGTTGGCAG |
